# Detection of Abnormal Extracellular Matrix in the Interstitium of Regenerating Renal Tubules

**DOI:** 10.3390/ijms151223240

**Published:** 2014-12-15

**Authors:** Will W. Minuth, Lucia Denk

**Affiliations:** Molecular and Cellular Anatomy, University of Regensburg, University Street 31, D-93053 Regensburg, Germany; E-Mail: lucia.denk@vkl.uni-regensburg.de

**Keywords:** kidney, stem/progenitor cells, abnormal development, extracellular matrix, glutaraldehyde, cupromeronic blue, ruthenium red, tannic acid, transmission electron microscopy

## Abstract

Stem/progenitor cells are promising candidates for the regeneration of parenchyma in acute and chronic renal failure. However, recent data exhibit that survival of stem/progenitor cells after implantation in diseased renal parenchyma is restricted. To elaborate basic parameters improving survival, cell seeding was simulated under advanced *in vitro* conditions. After isolation, renal stem/progenitor cells were mounted in a polyester interstitium for perfusion culture. During generation of tubules, chemically defined CO_2_ Independent Medium or Leibovitz’s L-15 Medium was applied. Specimens were then fixed for transmission electron microscopy to analyze morphological features in generated tubules. Fixation in conventional glutaraldehyde (GA) solution shows development of tubules each exhibiting a polarized epithelium, an intact basal lamina and an inconspicuous interstitium. In contrast, special fixation of specimens in GA solution containing cupromeronic blue, ruthenium red or tannic acid unveils previously not visible extracellular matrix. Control experiments elucidate that a comparable extracellular matrix is not present in the interstitium of the matured kidney. Thus, generation of renal tubules in combination with advanced fixation of specimens for electron microscopy demonstrates that development of abnormal features in the newly developed interstitium has to be considered, when repair of renal parenchyma is performed by implantation of stem/progenitor cells.

## 1. Introduction

In the last few years, numerous investigations were performed dealing with an implantation of stem/progenitor cells for the repair of diseased renal parenchyma [1,2,3]. From a theoretical point of view, the ability of stem/progenitor cells for self-renewal and the assumption that they develop functional parenchyma appear as ideal requirements to administer them for therapeutic regeneration [4]. However, from a practical standpoint one has to realize that after a currently performed implantation technique only a low percentage of cells survives [5]. In this respect, it makes no difference whether the applied technique for implantation is the infusion of stem/progenitor cells via the blood vessel system, the casual injection into diseased parenchyma or a subcapsular administration [6].

The minimal survival of stem/progenitor cells implanted in diseased renal parenchyma must have special causes. One of the crucial points is the period between a performed implantation and the initial repair within diseased parenchyma. During this phase of seeding, the promoting atmosphere for stem/progenitor cells co-implanted with a culture medium is replacing against harmful interstitial fluid of surrounding renal parenchyma [7,8]. Thus, to prevent damage, the idea is to implant stem/progenitor cells not in isolated form but embedded in a pad containing an artificial interstitium and a protecting culture medium [9].

The fact is that to date only little basic information about cell seeding of implanted stem/progenitor cells is available. Further on, the status of a pad construction is still far from implantation in animals. For that reason, in the present investigation an advanced culture model was applied [10]. To obtain first insights in parameters that may support seeding in the present experiments, stem/progenitor cells were isolated and mounted in a polyester interstitium for the generation of tubules. During perfusion culture, always fresh and chemically defined culture media were applied. While in previous experiments the effect of Iscove’s Modified Dulbecco’s Medium [11] on development of stem/progenitor cells was elaborated, in present experiments the influence of pH stabilized CO_2_ Independent Medium, and Leibovitz’s L-15 Medium was analyzed [12]. The focus was directed to morphological features arising in generated tubules. The actual data show that fixation of specimens in glutaraldehyde solution containing cupromeronic blue, ruthenium red or tannic acid for transmission electron microscopy unmasks previously not visible structures [13]. Based on these observations, for the first time causes for abnormal extracellular matrix in the interstitium of generated tubules can be systematically investigated.

## 2. Results and Discussion

The aim of the present investigation was to analyze by morphological techniques cell features and extracellular matrix of renal tubules generated within a polyester fleece by continuous application of pH stabilized CO_2_ Independent Medium or Leibovitz’s L-15 Medium.

### 2.1. Extension of Tubules within an Artificial Interstitium

**Figure 1:**
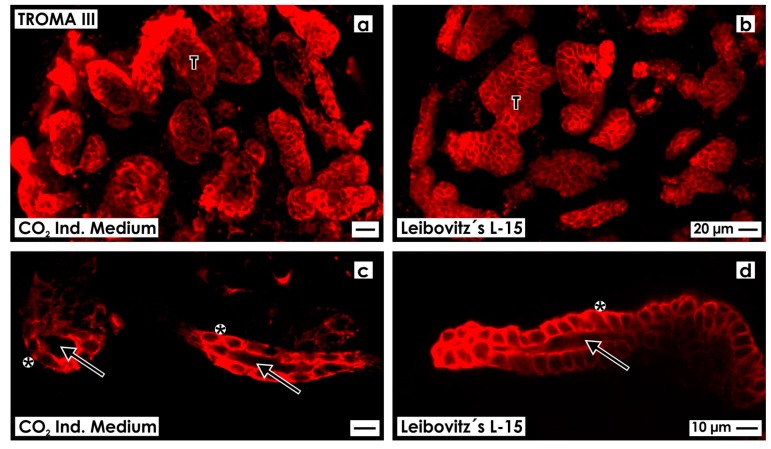
Confocal fluorescence microscopy of anti-TROMA III labeled renal tubules generated in an artificial interstitium during perfusion culture. Development of numerous tubules (T) can be seen, when CO_2_ Independent Medium (**a**) or Leibovitz’s L-15 Medium (**b**) was administered; (**c**,**d**) Analyzed tubules exhibit a lumen (arrow) and a basal lamina (asterisk).

After 13 days of perfusion culture, the artificial interstitium was opened by tearing off the fleece layers so that the threedimensional pattern of generated tubules could be analyzed by confocal fluorescence microscopy (Figure 1).

The area for tubule development within the artificial interstitium was 5 mm in diameter and up to 250 μm in height. When whole mount label by anti-TROMA III (anti-Cytokeratin 19) was performed, numerous tubules lining between fibers of the polyester fleece can be seen in a length-, oblique- and crosswise perspective (Figure 1a,b). Higher magnification illustrates that in generated tubules a single layer epithelium is contained forming a lumen and a basal lamina (Figure 1c,d). Comparing series generated in CO_2_ Independent Medium (Figure 1a,c) with series raised in Leibovitz’s L-15 Medium (Figure 1b,d), no morphological differences can be seen.

### 2.2. Extracellular Matrix in the Interstitium of Generated Tubules

**Figure 2:**
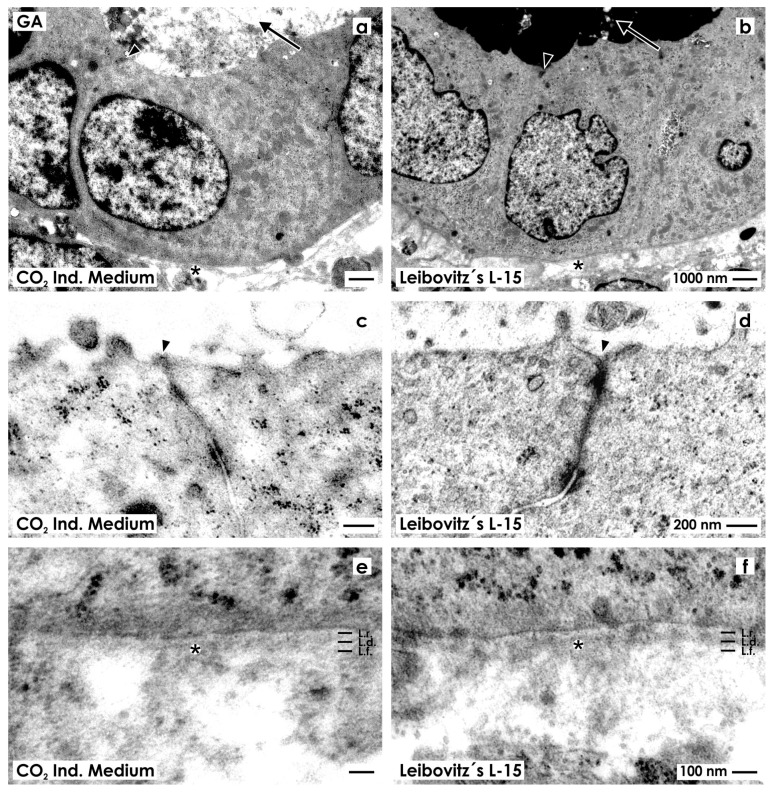
Transmission electron microscopy of renal tubules generated in CO_2_ Independent Medium (**a**,**c**,**e**) or Leibovitz’s L-15 Medium (**b**,**d**,**f**) after fixation in conventional glutaraldehyde (GA) solution. (**a**,**b**) Generated tubules show a polarized epithelium with a lumen (arrow) and a basal lamina (asterisk); (**c**,**d**) Between the luminal and lateral plasma membranes a tight junction (arrow head) is established; (**e**,**f**) In both cases the established basal lamina consists of a lamina rara (L.r.), lamina densa (L.d.) and lamina fibroreticularis (L.f.). The interstitium is bright and looks inconspicuously.

**Figure 3:**
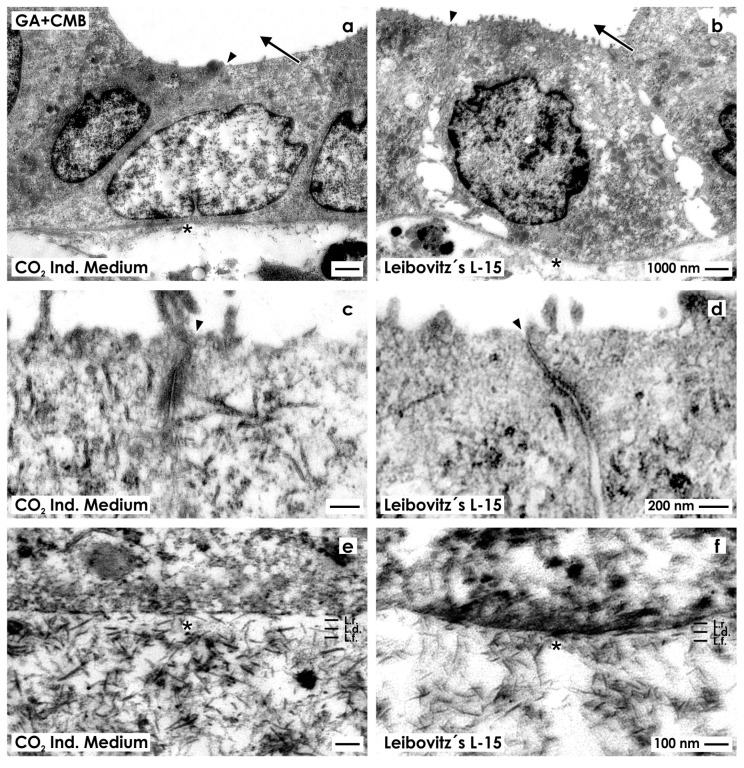
Transmission electron microscopy of renal tubules generated in CO_2_ Independent Medium (**a**,**c**,**e**) or Leibovitz’s L-15 Medium (**b**,**d**,**f**) after fixation in GA solution containing cupromeronic blue (CMB). (**a**,**b**) Generated tubules show a polarized epithelium with a lumen (arrow) and a basal lamina (asterisk); (**c**,**d**) Between the luminal and lateral plasma membranes a tight junction (arrow head) is established; (**e**,**f**) The basal lamina consists of a lamina rara (L.r.), lamina densa (L.d.) and lamina fibroreticularis (L.f.). Within the interstitium numerous braces of proteoglycans are seen that are linked to each other.

**Figure 4:**
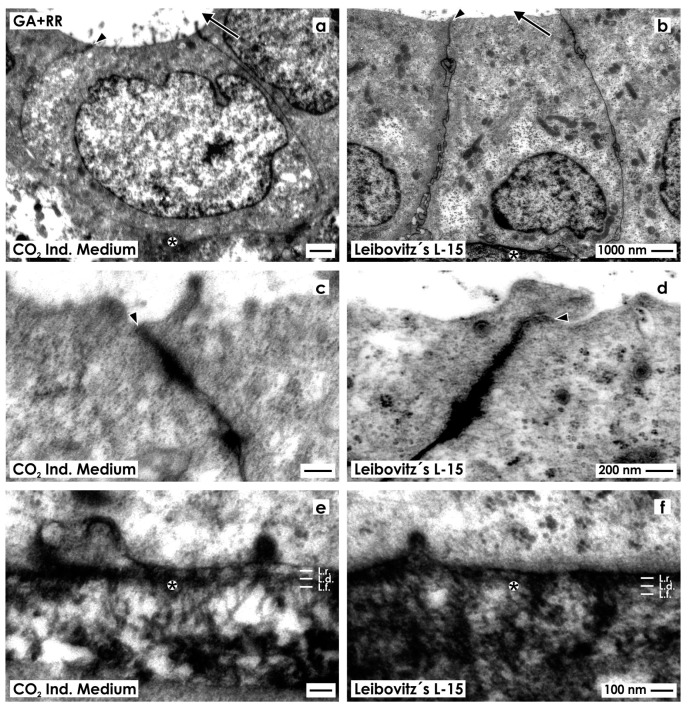
Transmission electron microscopy of renal tubules generated in CO_2_ Independent Medium (**a**,**c**,**e**) or Leibovitz’s L-15 Medium (**b**,**d**,**f**) after fixation in GA solution containing ruthenium red (RR). (**a**,**b**) Generated tubules show a polarized epithelium with a lumen (arrow) and a basal lamina (asterisk); (**c**,**d**) Between the luminal and lateral plasma membranes a tight junction (arrow head) is established; (**e**,**f**) The basal lamina consists of a lamina rara (L.r.), lamina densa (L.d.) and lamina fibroreticularis (L.f.). At the interstitium numerous fibers are forming a wide network.

**Figure 5:**
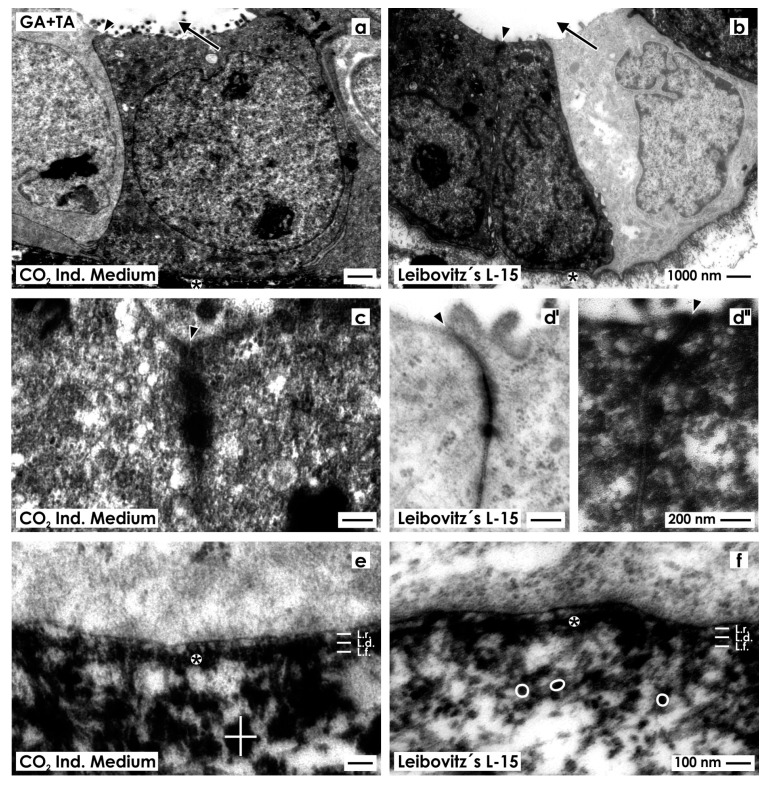
Transmission electron microscopy of renal tubules generated in CO_2_ Independent Medium (**a**,**c**,**e**) or Leibovitz’s L-15 Medium (**b**,**d**,**f**) after fixation in GA solution containing tannic acid (TA). (**a**,**b**) Generated tubules show a polarized epithelium with a lumen (arrow) and a basal lamina (asterisk). In contrast to previous series light and dark cells can be seen; (**c**,**d**) Between the luminal and lateral plasma membranes a typical tight junction (arrow head) is established; (**e**,**f**) The basal lamina consists of a lamina rara (L.r.), lamina densa (L.d.) and lamina fibroreticularis (L.f.). At the interstitium fibers are linked to each other via massive (white cross; diameter 150 nm), respectively, small (round white label; diameter 36 nm) thickenings resulting in a network.

**Figure 6:**
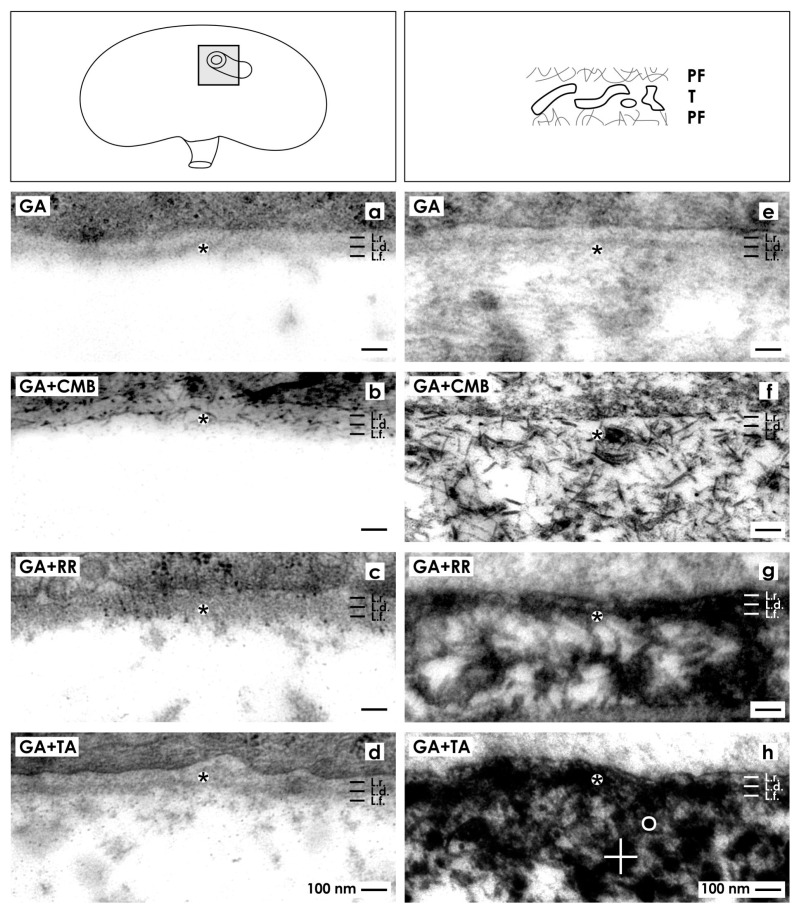
Electron microscopy in matured kidney (**a**–**d**) and generated tubules (**e**–**h**). In the kidney an unobtrusive interstitium (asterisk) is seen after fixation in GA solution (**a**); GA solution containing cupromeronic blue (**b**), ruthenium red (**c**) or tannic acid (**d**). Generated tubules exhibit after fixation in GA solution (**e**) an inconspicuous interstitium, while GA solution including cupromeronic blue (**f**), ruthenium red (**g**) or tannic acid (**h**) unveils extracellular matrix. Label by tannic acid shows fibers linked via massive (white cross; diameter 150 nm) or small (round white label; diameter 36 nm) thickenings.

Molecular interactions between an epithelium and the adjacent interstitium regulate tubulogenesis, maintainance of physiological functions and progess of parenchymal disease [14,15]. To access detailed morphological information about this important site, analysis by transmission electron microscopy was performed (Figure 2, Figure 3, Figure 4, Figure 5 and Figure 6).

In these series of experiments, specimens fixed in conventional glutaraldehyde solution were compared with specimens fixed in advanced solutions improving the contrast of compounds in the interstitium. Further on, to analyze comparable perspectives in transmission electron microscopy, all of the specimens were specifically orientated during embedding and sectioning. For documentation, always a surface view, high magnification of a tight junction and the basal aspect of a generated tubule cell are given.

#### 2.2.1. Fixation with Conventional Glutaraldehyde Solution

For control, fixation of specimens was performed in the first series with conventional GA solution.

Tubules generated in CO_2_ Independent Medium (Figure 2a) as in Leibovitz’s L-15 Medium (Figure 2b) illustrate epithelia with cuboidal cells. Inside of each cell, a nucleus with occasional folds is found. The cytoplasm is light and surprisingly few lysosomes can be seen. The luminal side of cells borders a lumen.

High magnification depicts that in both series neighboring epithelial cells are connected by a tight junction consisting of a zonula occludens, zonula adhaerens and a desmosome (Figure 2c,d). In all of the cases, a close basal slit is found at the transition between the lateral and basal plasma membranes indicating integrity of the epithelium.

In experiments with CO_2_ Independent Medium (Figure 2e) and Leibovitz’s L-15 Medium (Figure 2f), the basal aspect of cells rests on a basal lamina, which consists of a lamina rara, lamina densa and lamina fibroreticularis. Beyond the basal lamina, an explicit interstitial space is present. It looks bright, contains only few fibers of extracellular matrix and only some amorphous material. These morphological criteria point out that in generated tubules a polarized epithelium and an unobtrusively looking interstitium is established.

#### 2.2.2. Fixation with Glutaraldehyde Containing Cupromeronic Blue

To investigate more details of the interstitium and especially the occurrence of proteoglycans, in a next series of experiments generated tubules were fixed in GA solution containing cupromeronic blue (Figure 3).

Specimens raised as well in CO_2_ Independent Medium (Figure 3a) as Leibovitz’s L-15 Medium (Figure 3b) reveal that in tubules epithelia with cuboidal cells are developed. Each cell contains a nucleus with occasional folds. The cytoplasm appears bright and contains only few lysosomes. The luminal side of cells faces a lumen. High magnification depicts that in both series neighboring epithelial cells are connected by a tight junction consisting of a typical zonula occludens, zonula adhaerens and a desmosome (Figure 3c,d).

The basal aspect of epithelial cells rests on a basal lamina. Although barely visible as well, in experiments with CO_2_ Independent Medium (Figure 3e) as Leibovitz’s L-15 Medium (Figure 3f), it is registered that the newly developed basal lamina consists of a lamina rara, lamina densa and lamina fibroreticularis.

Further on, label by cupromeronic blue elucidates that the basal lamina is connected with the interstitium via numerous braces of proteoglycans linked with each other. This assembly forms a broad network. Most interestingly, the number of proteoglycan braces is slightly increased in series raised by CO_2_ Independent Medium (Figure 3e) as compared to series raised by Leibovitz’s L-15 Medium (Figure 3f).

#### 2.2.3. Fixation with Glutaraldehyde Containing Ruthenium Red

In a next series, specimens were fixed in GA solution containing ruthenium red to enhance the contrast of extracellular matrix in transmission electron microscopy. Samples raised in CO_2_ Independent Medium (Figure 4a) or Leibovitz’s L-15 Medium (Figure 4b) elucidate that in generated tubules a polarized epithelium with cuboidal cells is contained. The nuclei show occasional folds. In both cases, the cytoplasm is bright and contains only few lysosomes. The luminal side of cells faces a lumen.

High magnification illustrates that neighboring cells are connected by a tight junction consisting of a zonula occludens, zonula adhaerens and a desmosome (Figure 4c,d). In contrast to previous series, (Figure 2c,d and Figure 3c,d) this special site is yet intensively labeled by ruthenium red.

As well in series with CO_2_ Independent Medium (Figure 4e) as Leibovitz’s L-15 Medium (Figure 4f) the basal side of epithelial cells rests on a basal lamina. It consists of a lamina rara, lamina densa and lamina fibroreticularis. In addition, numerous fibers lining from the lamina fibroreticularis to the interstitium are intensively labeled by ruthenium red. In series raised by CO_2_ Independent Medium (Figure 4e) less fibers are seen, while in series generated with Leibovitz’s L-15 Medium (Figure 4f) much more fibers can be recognized forming a dense network.

#### 2.2.4. Fixation with Glutaraldehyde Containing Tannic Acid

In the following series, generated tubules were fixed in GA solution containing tannic acid to enhance contrast of extracellular fibrils. Samples raised in CO_2_ Independent Medium (Figure 5a) or Leibovitz’s L-15 Medium (Figure 5b) show an epithelium with cuboidal cells. Inside the cells, a nucleus with occasional folds is present.

In contrast to previous series (Figure 2a,b, Figure 3a,b and Figure 4a,b), the epithelium shows after fixation in GA solution containing tannic acid a heterogeneously composed cell population as it was earlier described [13]. The one type of cells exhibits a bright cytoplasm, while the other type shows a dark-grey cytoplasm. In both of the cases, the cytoplasm of cells contains only few lysosomes. The luminal side of cells faces a lumen.

High magnification illustrates that in neighboring epithelial cells a tight junction is established consisting of a typical zonula occludens, zonula adhaerens and a desmosome intensively labeled by tannic acid (Figure 5c,d',d'').

The basal side of epithelial cells rests on a basal lamina. In series with CO_2_ Independent Medium (Figure 5e) or Leibovitz’s L-15 Medium (Figure 5f), it consists of a lamina rara, lamina densa and lamina fibroreticularis. Numerous fibers labeled by tannic acid line from the lamina densa to the interstitium. In series raised with CO_2_ Independent Medium fibers are observed that show massive thickenings (Figure 5e; see label by a white cross; diameter 150 nm). In contrast, series generated with Leibovitz’s L-15 Medium exhibit a network of fibers linked via small thickenings (Figure 5f; round white label; diameter 36 nm).

#### 2.2.5. Advanced Fixation in Matured Kidney Parenchyma *versus* Generated Tubules

In the next series was investigated whether unveiled extracellular matrix in generated tubules can be also detected in the kidney. Thus, in this coherence, it must be considered that presently applied protocols of fixation with GA solution containing cupromeronic blue (Figure 3e,f), ruthenium red (Figure 4e,f) or tannic acid (Figure 5e,f) for generated tubules may unveil in the kidney an analogous contrast pattern.

In order to check this assumption, matured renal parenchyma was fixed in a control series with conventional GA solution (Figure 6a) and compared with generated tubules (Figure 6e). It can be seen that the interstitium within the kidney looks fully unobtrusive (Figure 6a). In the interstitium of generated tubules, tiny fibers and some fuzzy material is recognized (Figure 2a,b and Figure 6e). Renal parenchyma fixed in GA solution containing cupromeronic blue shows only few braces of proteoglycans in the basal lamina of tubules, while the interstitium is bright (Figure 6b). In contrast, generated tubules exhibit in their basal lamina and especially in the interstitium numerous braces of proteoglycans (Figure 3e,f and Figure 6f). Further on, matured kidney fixed in GA solution containing ruthenium red shows only a weak label on the basal lamina, while the adjacent interstitial space is bright (Figure 6c). However, generated tubules demonstrate that numerous intensively labeled fibers are contained in the interstitium (Figure 4e,f and Figure 6g).

Finally, renal parenchyma fixed in GA solution containing tannic acid shows an inconspicuous basal lamina and a bright interstitium with few fuzzy material (Figure 6d). In contrast, generated tubules demonstrate that the newly developed interstitium is filled with numerous black labeled fibers linked to each other via thickenings (see label by a cross; diameter 150 nm *versus* round label; diameter 36 nm) (Figure 5e,f and Figure 6h).

In conclusion, this last set of experiments reveals that an up to date unknown interstitial matrix labeled by cupromeronic blue (Figure 3e,f and Figure 6f), ruthenium red (Figure 4e,f and Figure 6g) and tannic acid (Figure 5e,f and Figure 6h) is synthesized by generated tubules that cannot be detected in the kidney (Figure 6b–d).

### 2.3. Unveiling of Extracellular Matrix by Advanced Fixation

It is well known that the interstitium plays a crucial role in controlling of stem/progenitor cell properties and development of tubules [16,17,18]. Analytical focus in the present set of experiments was therefore directed to morphological features in the newly formed interstitium of generated tubules.

It is common practice to fix species in conventional GA solution for analysis in transmission electron microscopy. Following this concept, the interstitium looks bright and only occasionally can be seen that fibers of extracellular matrix traverse the interstitium (Figure 2e,f and Figure 6e). In contrast, when fixation of specimens is performed in GA solution containing cupromeronic blue, numerous braces of proteoglycans become visible that are linked between themselves forming in turn a network (Figure 3e,f and Figure 6f). In addition, fixation of specimens in GA solution containing ruthenium red unveils in the interstitium of generated tubules numerous fibers that are linked with each other (Figure 4e,f and Figure 6g). However, the matrix labeled by ruthenium red is not identical with braces of proteoglycans labeled by cupromeronic blue. Finally, fixation of specimens in GA solution containing tannic acid reveals an additional textural pattern of fibers within the interstitium of generated tubules (Figure 5e,f and Figure 6h).

The present data clearly point out that fixation of specimens in conventional GA solution (Figure 2e,f and Figure 6e) shows only a minimal fraction of extracellular matrix that becomes visible after fixation of specimens by GA solution containing cupromeronic blue (Figure 3e,f and Figure 6f), ruthenium red (Figure 4e,f and Figure 6g) or tannic acid (Figure 5e,f and Figure 6h). Due to the lack of sufficient contrast data raised from stem/progenitor cell preparations fixed solely by conventional GA solution for transmission, electron microscopy must be more critically analyzed in future.

### 2.4. Recognition of Abnormal Features in Regenerating Tubules

Fixation of renal parenchyma as well in conventional GA solution (Figure 6a) as GA solution containing cupromeronic blue (Figure 6b), ruthenium red (Figure 6c) or tannic acid (Figure 6d) reveals an interstitium that looks unobtrusive. Also, generated tubules fixed in conventional GA solution demonstrate barely recognizable extracellular matrix in the interstitium. In contrast, fixation of generated tubules in GA solution containing cupromeronic blue (Figure 3e,f and Figure 6f), ruthenium red (Figure 4e,f and Figure 6g) or tannic acid (Figure 5e,f and Figure 6h) illustrates previously not visible structures in the newly developed interstitium.

Since a comparable extracellular matrix is not contained within intact kidney parenchyma (Figure 6a–d), newly detected extracellular matrix has to assigned to abnormal development in generated tubules (Figure 3e,f, Figure 4e,f, Figure 5e,f and Figure 6f–g). The here presented results of abnormal development supplement earlier findings. In those previous experiments, it was demonstrated for example that application of aldosterone (concentration 1 × 10^−7^ M) resulted in formation of intact tubules, while treatment with pregnenolone, 11-desoxycorticosterone or corticosterone caused abnormal development of cell islets respective of cell clusters [19,20].

Thus, earlier and present findings clearly point out that regeneration induced by stem/progenitor cells results in tubule formation but it does not happen without biomedical risk. As a consequence, one has to accept that development of renal stem/progenitor cells is much more sensible as earlier believed and must be investigated therefore more critically in future.

## 3. Experimental Section

### 3.1. Isolation of Renal Stem/Progenitor Cells

The kidneys of one-day old narcotised and sacrificed New Zealand rabbits were isolated and cut into two parts as it was earlier described [11]. Stripping off the capsula fibrosa with fine forceps a thin layer of stem/progenitor cell niches adheres to the explant. Applying this simple isolation method a layer of up to 1 cm^2^ in square can be harvested for subsequent cell biological analysis.

### 3.2. Artificial Interstitium for Perfusion Culture

**Figure 7:**
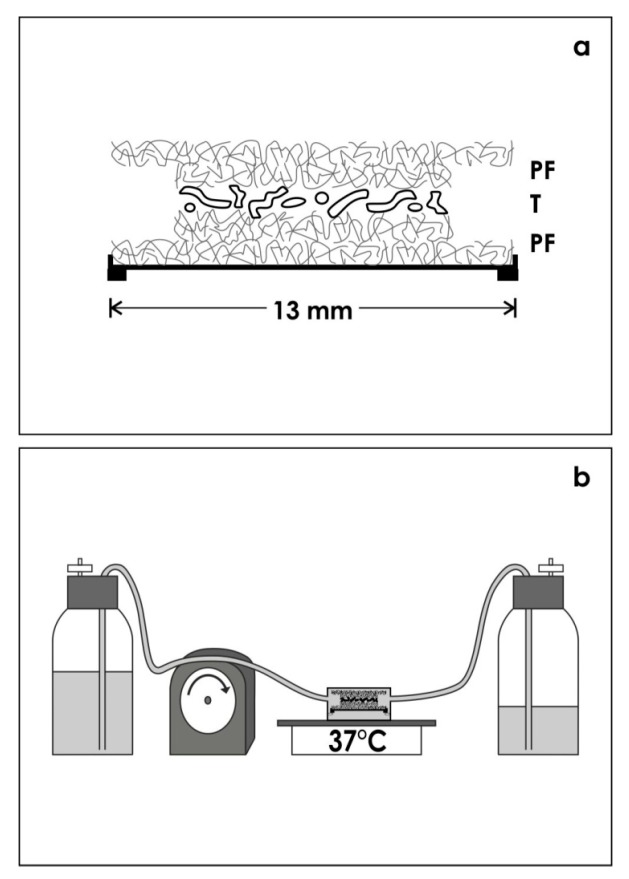
Schematic illustration shows generation of renal tubules within an artificial interstitium. (**a**) In present culture experiments, the isolated tissue layer containing renal stem/progenitor cells was placed between polyester fleeces and mounted in a MINUSHEET^®^ tissue carrier; (**b**) For subsequent culture, the tissue carrier was transferred to a perfusion culture container with horizontal flow characteristics. During a culture period of 13 days chemically defined culture medium was not re-circulated but transported from the storage to a waste bottle.

In present culture experiments, the isolated tissue layer was placed between punched out pieces of polyester fleece (I7, Walraf, Grevenbroich, Germany) as it was earlier described [11]. To prevent damage during culture, the sandwich was mounted in a MINUSHEET^®^ tissue carrier (Minucells and Minutissue, Bad Abbach, Germany) with 13 mm inner diameter (Figure 7a).

For subsequent culture, the tissue carrier was mounted in a perfusion container with horizontal flow characteristics (Minucells and Minutissue). To maintaining a constant temperature of 37 °C, the perfusion culture container was placed on a thermoplate (Medax-Nagel, Kiel, Germany) (Figure 7b). During a period of 13 days, always fresh medium was transported at a rate of 1.25 mL/h with an IPC N8 peristaltic pump (Ismatec, Wertheim, Germany).

To generate tubules chemically defined CO_2_ Independent Medium (Nr. 18045-054) or Leibovitz’s L-15 Medium (Nr. 31415-029) both including Phenolred (GIBCO/Invitrogen, Karlsruhe, Germany) were applied.

Infections in perfusion culture were prevented by adding an antibiotic-antimycotic cocktail (1%, GIBCO/Invitrogen). Tubulogenic development was induced by application of aldosterone (1 × 10^−7^ M, Fluka, Taufkirchen, Germany) as it was earlier described [19]. To reach a constant pH of 7.4 under atmospheric air *N*-2-hydroxyethylpiperazine-*N*-2-ethane sulfonic acid (HEPES; GIBCO/Invitrogen) was added by titration in necessary amounts.

### 3.3. Histochemistry

For whole mount label tubules generated within a polyester fleece as an artificial interstitium were fixed in 70% ethanol, washed several times with phosphate buffered saline (PBS) and incubated for 30 min in blocking solution (PBS, pH 7.5, 10% horse serum from GIBCO/Invitrogen, 1% bovine serum albumin from Serva, Heidelberg, Germany). Then, incubation for 1 hour was performed with undiluted monoclonal anti-TROMA III (anti-Cytokeratin 19, Developmental Studies of Hybridoma Bank, University of Iowa, Iowa City, IA, USA). After washing with 1% BSA in PBS the specimens were incubated for 45 minutes with goat-anti-rat-IgG-rhodamine (Jackson Immunoresearch Laboratories, West Grove, PA, USA) diluted 1:50 in PBS containing 1% BSA.

Following several washes with PBS the specimens were analyzed using a CM12 confocal laser scanning microscope (Zeiss, Oberkochen, Germany). Fluorescence images were taken with a digital camera at a standard exposure time and thereafter processed with Corel DRAW Graphic Suite X5 (Corel Corporation, Otawa, ON, Canada).

### 3.4. Fixation and Embedding

Generated tubules and intact renal parenchyma were transferred to immersion fixation for transmission electron microscopy (TEM). Following solutions were used:

- Series **1**: 5% glutaraldehyde (Serva) buffered with 0.15 M sodium cacodylate, pH 7.4;
- Series **2**: 5% glutaraldehyde buffered with 0.15 M sodium cacodylate, pH 7.4. Then incubation was performed with 0.1% cupromeronic blue (Santa Cruz, Heidelberg, Germany) and 0.1 M magnesium chloride hexahydrate (Sigma, Taufkirchen, Germany) in sodium acetate buffer, pH 5.6. Counterstain was performed with 0.5% sodium tungstate dehydrate (Sigma);
- Series **3**: 5% glutaraldehyde buffered with 0.15 M sodium cacodylate, pH 7.4 containing 0.5% ruthenium red (Fluka);
- Series **4**: 5% glutaraldehyde buffered with 0.15 M sodium cacodylate, pH 7.4 containing 1% tannic acid (Sigma).

Fixation was made for 1 day at room temperature. After several washes with 0.15 M sodium cacodylate the specimens except the ones with cupromeronic blue were postfixed in the same buffer but containing 1% osmium tetroxide (Science Services, München, Germany).

Before embedding all of the specimens were washed with sodium cacodylate buffer and dehydrated in graded series of ethanol.

Finally, the specimens were embedded in Epon (Fluka), which was polymerized at 60°C for 48 h.

### 3.5. Positioning of Tissue for Morphological Analysis

For first orientation, semithin sections of specimens were performed as it was earlier described [13].

**Figure 8:**
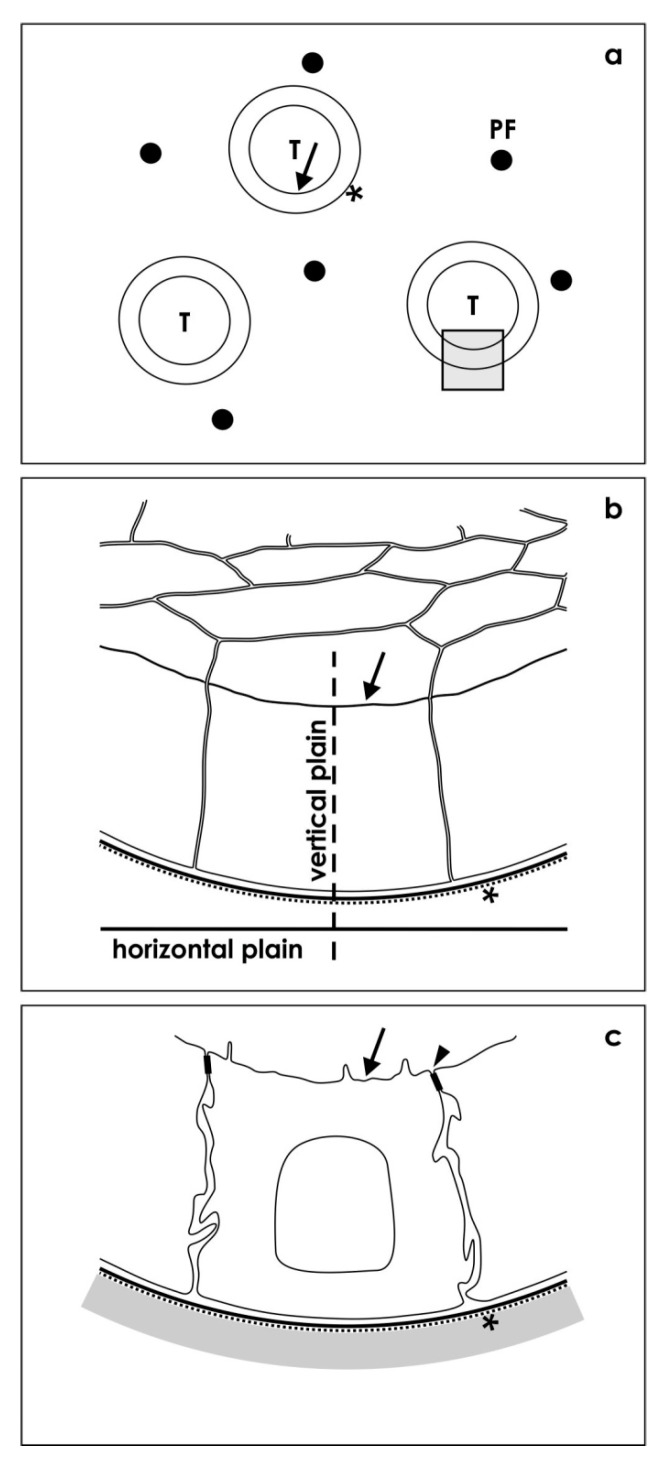
Illustration depicts section plains of generated tubules. (**a**) By light microscopy first semithin sections of tubules (T) are screened occurring between polyester fibers (PF) of an artificial interstitium. Exact cross-sectioned tubules (insert) show a lumen (arrow) and a basal lamina (asterisk); (**b**) In electron microscopy, epithelia of selected tubules were analyzed in an exact vertical perspective; (**c**) Resulting sections’ respective illustrations show always the same perspective of epithelial cells with a lumen (arrow), a tight juntion (arrow head), a basal lamina (asterisk) and a neighboring interstitium.

To obtain a comparable view to different experimental series, by the light microscope exactly cross-sectioned tubules were identified (Figure 8a).

Most important, in the following step a resin block was trimmed so that ultrathin sections of this specific area could be performed. For this work, a diamond knife and an ultramicrotome EM UC6 (Leica, Wetzlar, Germany) was used. Resulting sections were collected onto slot grids coated with 1.5% Pioloform (Plano, Wetzlar, Germany) and contrasted using 1% uranyl acetate and lead citrate as it was earlier described [21].

### 3.6. Transmission Electron Microscopy (TEM)

Sections were examined at 80 kV using an EM 902 transmission electron microscope (Zeiss). Electron micrographs were recorded digitally using a slow scan CCD camera and thereafter processed with Adobe Photoshop (Adobe, San Jose, CA, USA) and Corel DRAW Graphic Suite X5 (Corel Corporation).

Only those tubules were analyzed showing a lumen and a basal lamina exactly cut in a vertical plain (Figure 8b).

When this strategy was followed, the epithelium of tubules is perfectly orientated for further analysis. As a consequence, all of the here demonstrated micrographs show this perspective allowing in turn comparisons between different experimental series (Figure 8c).

### 3.7. Amount of Analyzed Specimens

A total of 21 orientated tubule epithelia were analyzed for the present investigation.

## 4. Conclusions

The present data demonstrate that fixation of generated renal tubules in conventional GA solution for transmission electron microscopy shows only a minimal fraction of extracellular matrix that becomes visible after fixation of specimens by GA solution containing cupromeronic blue, ruthenium red or tannic acid. Further on, the here described interstitial matrix is exclusively synthesized during generation of tubules and cannot be detected in the matured kidney. It is obvious that described abnormal development must have special reasons. Therefore, molecular analysis of illustrated extracellular matrix is a current focus of ongoing research. Finally, data raised from stem/progenitor cell preparations fixed solely by conventional GA solution for transmission electron microscopy generally must be critically questioned. Hence, the present investigation shows that advanced protocols for fixation and contrasting of extracellular matrix are yet available.
